# Influence of hooks and a lag screw on internal fixation plates for lateral malleolar fracture: a biomechanical and ergonomic study

**DOI:** 10.1186/s13018-017-0537-8

**Published:** 2017-02-23

**Authors:** Rina Sakai, Masataka Uchino, Terumasa Yoneo, Yasuaki Ohtaki, Hiroaki Minehara, Terumasa Matsuura, Tsutomu Gomi, Masanobu Ujihira

**Affiliations:** 1 Department of Medical Engineering and Technology, Kitasato University, 1-15-1 Kitasato, Minami-ku, Sagamihara, Kanagawa 252-0373 Japan; 2 Department of Orthopedic Surgery, Kitasato University Medical Center, 6-100 Arai, Kitamoto, Saitama 364-8501 Japan; 3 Graduate School of Medical Sciences, Kitasato University, 1-15-1 Kitasato, Minami-ku, Sagamihara, Kanagawa 252-0373 Japan; 4 Faculty of Engineering, Kanagawa Institute of Technology, 1030 Shimo-Ogino, Atsugi, Kanagawa 243-0292 Japan; 5 Department of Orthopedic Surgery, Kitasato University, 1-15-1 Kitasato, Minami-ku, Sagamihara, Kanagawa 252-0373 Japan

**Keywords:** Osteosynthesis, Lateral malleolar fractures, Internal fixation, One-third tubular plate, Locking compression plate

## Abstract

**Background:**

For internal fixation of AO classification Type B lateral malleolar fracture, insertion of lag screws into the fracture plane and fixation with a one-third tubular plate as a neutralization plate are the standard treatment procedures. The one-third tubular plate is processed to a hook shape and hung on the distal end of the fibula. In this study, to compare the function of the hook and lag screws of a one-third tubular plate and LCP for osteosynthesis of lateral malleolar fracture, mechanical indices of internal fixation were compared among the one-third tubular plates with lag screws with and without the hook and a locking compression plate.

**Methods:**

As mechanical tests, a compression test was performed in which compression in the bone axis direction produced by supporting the body weight was simulated, and a torsion test was performed in which external rotation of the bone axis caused by plantar flexion of the ankle joint was simulated. Muscle strength during walking and the force and torque acting on the ankle and knee joints were determined using inverse dynamic analysis. Finite element analysis was performed to analyze the function of hooks and lag screws. The joint reaction force determined by inverse dynamic analysis was adopted as the loading condition of finite element analysis.

**Results:**

A stiffness equivalent to that of healthy bone could be achieved by all three internal fixations. It was clarified that the presence of the hook does not make a difference in stiffness. Displacement of the one-third tubular plate was small regardless of the presence or absence of the hook compared with those of locking compression plates.

**Conclusions:**

The presence of the hook did not make any difference in stiffness, suggesting that active preparation of the hook is unnecessary. We also clarified that lag screws inhibit displacement.

## Background

The ankle joint can be damaged by rotation and lateral or longitudinal external forces, which in many cases cause subluxation of the talus and malleolar fractures. The aim of treatment for malleolar fractures is to accurately reduce the fracture and ensure sufficient stability to allow early movement [1]. Anatomical reduction and stable fixation of unstable fractures with dislocation are provided by open reduction and internal fixation (ORIF) [2–4]. The characteristic patterns of malleolar fracture have been classified. The Association for Osteosynthesis/Orthopaedic Trauma Association (AO/OTA) classification, based on that by Weber in 1972, classifies trans-syndesmotic fibular fracture as Type B [1, 5]. Type B is the most common fracture; in many cases, the fracture is oblique in the anteroinferior to posterosuperior direction [6]. Internal fixation is generally achieved by insertion of cortical bone lag screws into the fracture surface and the use of a one-third tubular plate adjusted to the shape of the lateral surface of the fibula as a neutralization plate [1].

The one-third tubular plate has the form of one-third of the circumference of a cylinder. Favorable treatment outcomes have occasionally been reported by processing the cut end to a hook shape and then applying the hook to enclose the distal end of the fibula [7, 8]. Although these clinical reports suggest the value of the hook in this use, basic data to support this use have not been obtained.

In this study, we compared the function of the hook and lag screws of a one-third tubular plate with that of a locking compression plate (LCP) for osteosynthesis of lateral malleolar fracture using mechanical indices of internal fixation by comparing one-third tubular plates with lag screws with and without a hook and a locking compression plate. In addition to the biomechanical investigation, we also conducted an ergonomic investigation of the function of internal fixation plates, which is unique to this study. Although various clinical reports, surgical techniques, and biomechanical studies have described aspects of lateral malleolar fracture, this is the first ergonomic study to quantitatively measure weights loaded on the implant in consideration of the muscles and skeleton around the ankle joint [9–12].

## Methods

This study was approved by the Ethics Committee of Kitasato University School of Allied Health Sciences.

### Biomechanical study

**Fig. 1:**
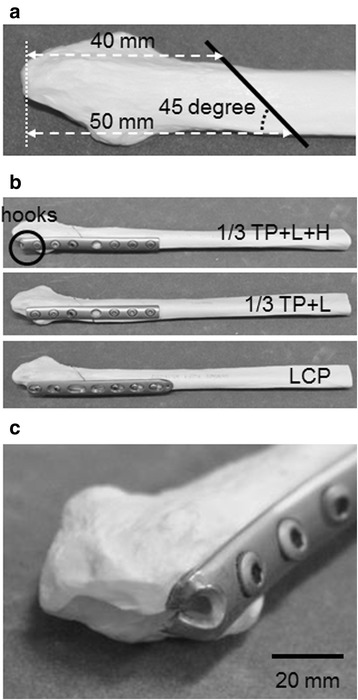
Specimens. **a** Fracture line of fibula (*solid black line*). **b** Three internal fixations. **c** Enlarged image of hooks on a 1/3 TP + L + H

The test material was a simulated resin fibula (Distal Fibula, SAWBONES, WA, USA). The fracture line was made uniform in all cases by simulating a simple AO classification Type B fracture (Fig. 1a) [13]. The simulated fibula without a fracture line was used as control. We investigated three fixation cases: a one-third tubular plate with lag screws and a prepared hook (1/3 TP + L + H) (DePuy Synthes, Japan), a one-third tubular plate with lag screws without a hook (1/3 TP + L) (DePuy Synthes, Japan), and LCP (DePuy Synthes, Japan) (Fig. 1b). The three types of internal fixation were applied to the lateral fibula following the standardized procedure. The hook region of 1/3 TP + L + H was prepared by cutting from the distal end of the plate up to the eighth (final) hole at two sites and bending the resulting forks inward by 90° (Fig. 1c). At fixation, a lag screw was first placed in the third hole from the distal end perpendicular to the fracture line, and the hook was then shaped by hammering to conform to the distal end of the fibula. The plate was then tightened to the bone with two and three screws (3.5 mm diameter) at the distal and proximal sides of the plate, respectively. In fixation with 1/3 TP + L, after fixation with lag screws, the plate was tightened to the bone with two and three screws at the distal and proximal sides of the plate, respectively. In fixation with LCP, no lag screw was used, and the plate was tightened to the bone with two and three locking screw screws (3.5 mm diameter) at the distal and proximal sides, respectively.

In mechanical testing, a compression test simulated the compression along the longitudinal bone axis direction produced by supporting the body weight, and a torsion test simulated the external rotation of the bone axis caused by plantar flexion of the ankle joint [14]. For the compression test, a load testing machine (FGS-50VB-L, SHIMPO, Japan) and digital load sensor (FGP-100, SHIMPO, Japan) were used. The load sensor was moved from the upper to the lower sides at a speed of 10 mm/min to apply a load on the proximal end of the fibula. Displacement of the fibula was extracted from the tester, the load value was extracted from the load sensor, and a load-displacement curve was prepared. Regarding the maximum detected load as the compressive strength, the slope of the linear region of the curve between 100 and 200 N was defined as compressive stiffness. In the torsion test, a robot arm (RV-M2, Mitsubishi Electric, Japan) and six-shaft strain gage-type load transducer (CA95776 JR3, NC, USA) were used. The distal end of the arm was externally rotated by 30°. The produced torque was measured using the load transducer and divided by the rotation angle to determine torsional stiffness. In statistical analysis, one-way layout analysis of variance and multiple comparisons employing the Tukey-Kramer method were performed.

### Ergonomic study

**Fig. 2:**
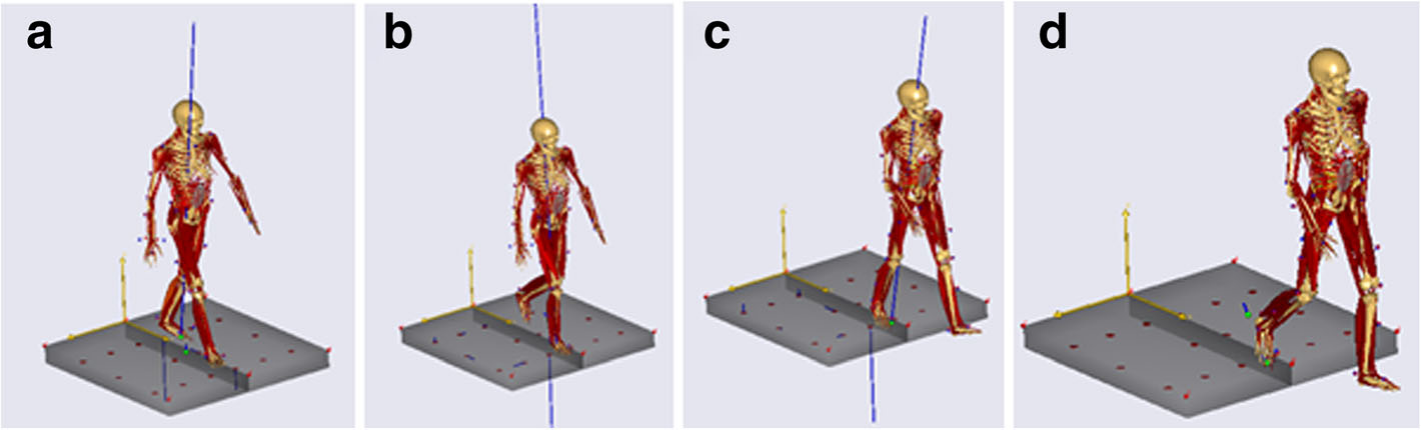
Sequence walking. **a** Initial contact. **b** Mid stance. **c** Terminal stance. **d** Pre-swing

Using an optical motion capture system, Vicon Motion Systems VICON 512 (Vicon Motion Systems Ltd., UK), three-dimensional motions of gait were video-recorded. The position was measured from reflected infrared marker lights emitted by nine Progressive Scan CCD cameras (TM-6710, JAI PULiX Inc., CA, USA) and recorded at a measurement frequency of 120 Hz. At the same time, motion was recorded using an analog video camera (HSV-500C3, nac Image Technology Inc., Japan) at 30 frames/s. The subject was a healthy male in his 20s to whom 43 markers of the Vicon Plug-in-Gait marker set were applied according to the manufacturer’s instructions (Vicon Plug-in-Gait Manual, 2003). The subject walked on a force plate (Z15907A, KISTLER, Japan) five times (Fig. 2). The floor reaction force was measured using two force plates and eight-channel charge amplifiers (9865, KISTLER, Japan).

The range of motion of each joint and conversion to the skeleton model (Vicon Workstation 4.5 Build 124; Vicon Motion Systems Ltd., UK) was used, and the results were output as c3d files. The coordinate system and definitions of joint angles were established in accordance with the Plug-in-Gait protocol (Vicon Plug-in-Gait Manual, 2003), setting the baseline posture to the anatomical standing position. Muscle strength during walking and the force and torque acting on the ankle and knee joints were determined using inverse dynamic analysis. For software, AnyBody Modeling System ver. 6.0.4 (AnyBody Technology A/S, Denmark) was used [15, 16]. Twelve muscles, including 10 of the muscles attaching to the fibula and the short and long heads of biceps femoris, were analyzed (Table 1).

**Table 1:**
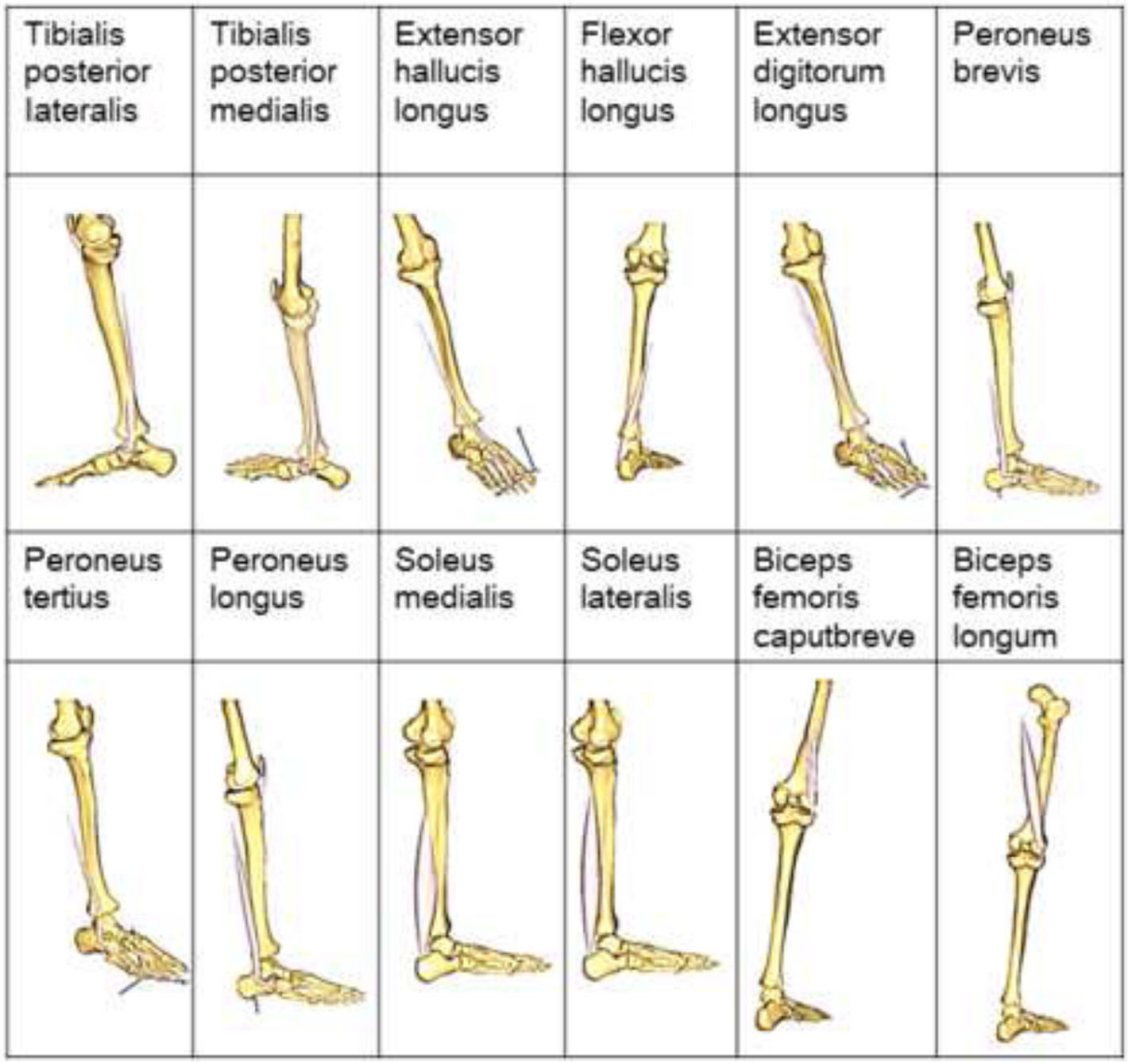
Target muscles in ergonomic analysis

### Finite element analysis

**Fig. 3:**
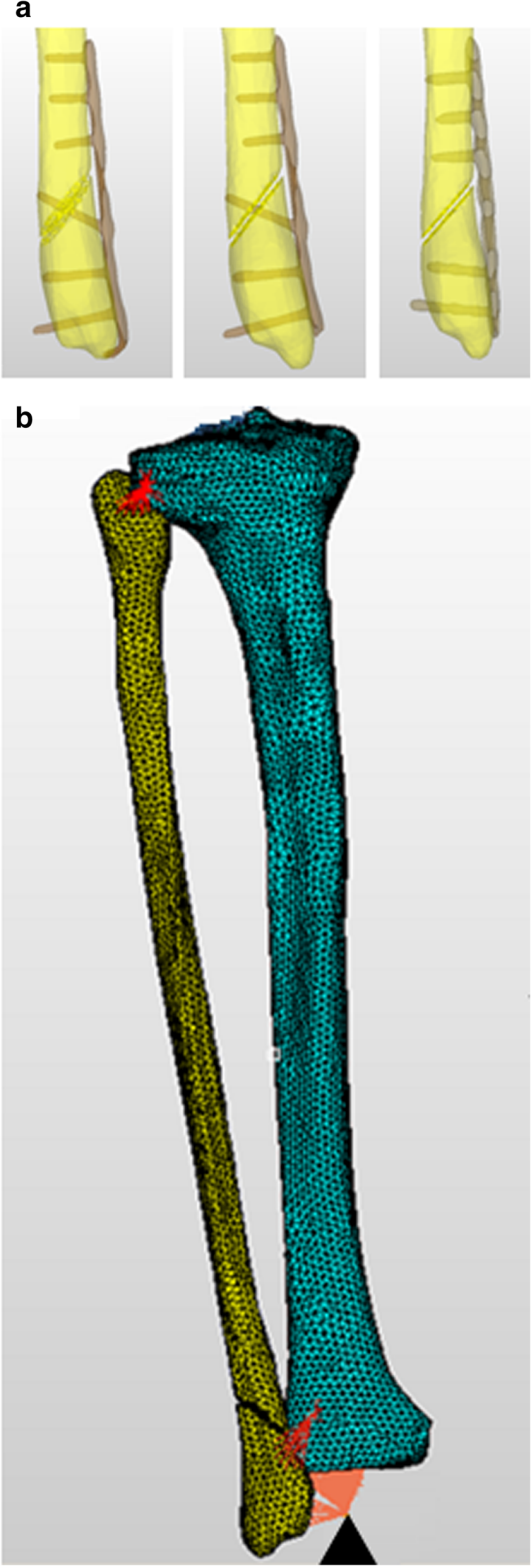
Finite element models. **a** Implant models of three internal fixations. 1/3 TP + L + H (*right*). 1/3 TP + L (*mid*). LCP (*left*). **b** Bone models of the fibula and tibia. The *vertex of a black triangle* is restriction. *Red lines* are binding parts

Finite element analysis (FEA) was performed to analyze the function of hooks and lag screws. Since AnyBody Modeling System ver. 6.0.4 does not incorporate the modeling of implants or the fibula, images of the simulated resin fibulas with the three types of internal fixation were acquired using μCT (inspeXio SMX-90CT, Shimazu, Japan) to prepare finite element models. Mesh models were prepared with tetrahedral elements from these images using Simpleware image processing software (Simpleware Ltd., UK). Each of the 1/3 TP + L + H, 1/3 TP + L, and LCP models were constructed with about 200,000 elements (Fig. 3a). For material constants, we defined elastic modulus, Poisson’s ratio, and mass density as 10 GPa, 0.3, and 2.0 g/cm^3^ for the fibula and tibia [17, 18] and 100 GPa, 0.3, and 4.5 g/cm^3^ for the plates, respectively [19, 20]. The muscles attaching to the fibula were regarded as stiff attachments of the tibia in the calculation. Since the origin and insertion of the muscles correspond to locations on the fibula, modeling of the fibula incorporated the muscle strengths at the origin and insertion.

Analysis was conducted using an Endeavor Pro-4500 (EPSON, Japan) and finite element analysis code LS-DYNA ver.971 (LSTC, CA, USA) as hardware and software, respectively. The constraint condition was set to integrity constraint centering on the ankle joint, and rigid connection was partially applied to the fibula and tibia (Fig. 3b). The joint reaction force determined by inverse dynamic analysis was adopted as the loading condition.

## Results

**Fig. 4:**
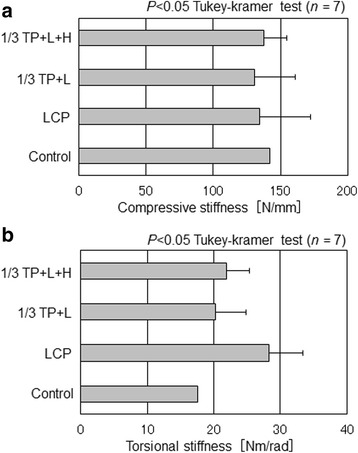
Stiffness measured by loading test of three internal fixations. **a** Compressive stiffness. **b** Torsional stiffness

No significant difference among the three internal fixations was noted in either the compressive or torsional stiffness test (*p* < 0.05), and all internal fixations achieved stiffness equivalent to that of the non-fractured simulated bone (control) (Fig. 4a), showing that the presence or absence of the hook did not make a difference in stiffness. Torsional stiffness of LCP was 25% higher than that of the one-third tubular plates, although the difference was not significant (Fig. 4b).

On the ankle joint, forces of 2008, 453, and 407 N were loaded on the proximal, anterior, and lateral regions, respectively. These forces reached maximum at the terminal stance (TS) and were second highest at mid stance (MS) (Table 2). When the knee joint reaction force was maximum, a weight about two times greater than the body weight was loaded on the ankle joint, and when ankle joint reaction force was maximum, a weight about three times greater than the body weight was loaded on the ankle joint. The maximum torque loaded on the ankle joint was 14 Nm at TS, followed by 11 Nm at MS. Torque measured in the biomechanical study was 14 Nm in the bone fixed with LCP and 11.5 Nm in the bone fixed with 1/3 TP + L + H, and equivalent to those in the ergonomic study.

**Table 2:**
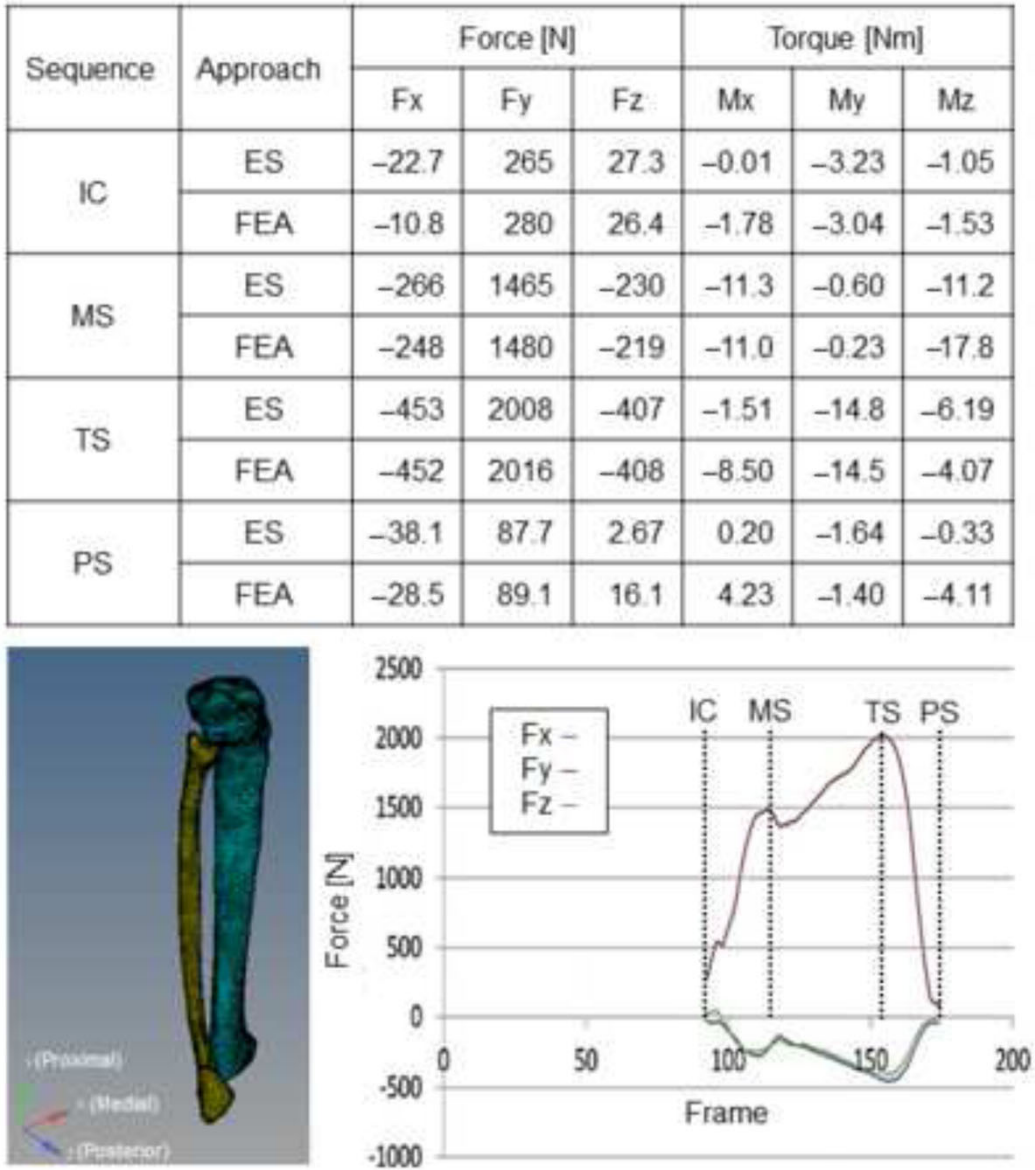
Force and torque loaded on the ankle joint in each sequence

The left lower image indicates the axial directions of the force and torque in the table. The right lower figure shows the force in each axial direction calculated by ergonomic study (ES). The dotted lines represent the numbers of frames in the sequences

**Fig. 5:**
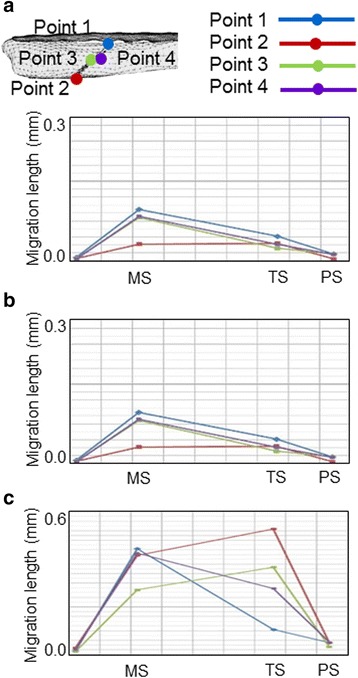
Migration length of four points on the fracture line. *Point 1*: fibula lateral. *Point 2*: fibula medial. *Point 3*: fibula anterior. *Point 4*: fibula posterior. **a** 1/3 TP + L + H. **b** 1/3 TP + L. (**c**) LCP

**Fig. 6:**
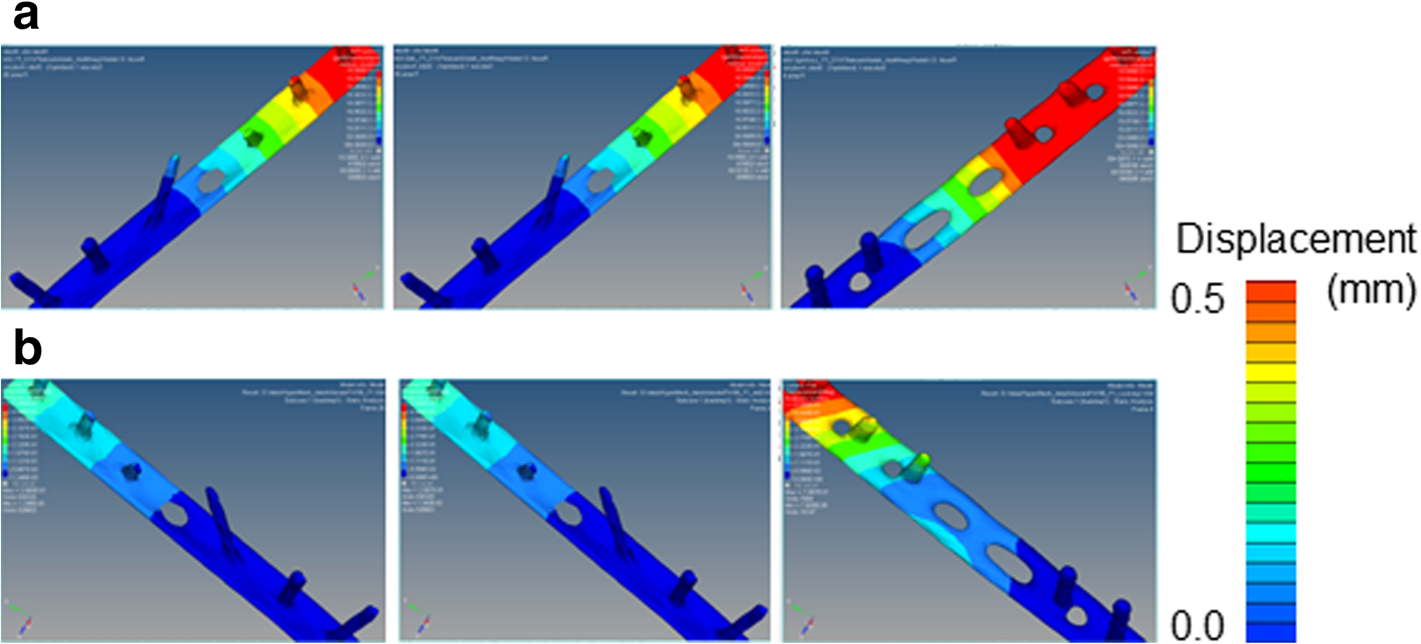
Displacement of three internal fixation models. 1/3 TP + L + H (*right*). 1/3 TP + L (*mid*). LCP (*left*). **a** Mid stance. **b** Terminal stance

**Fig. 7:**
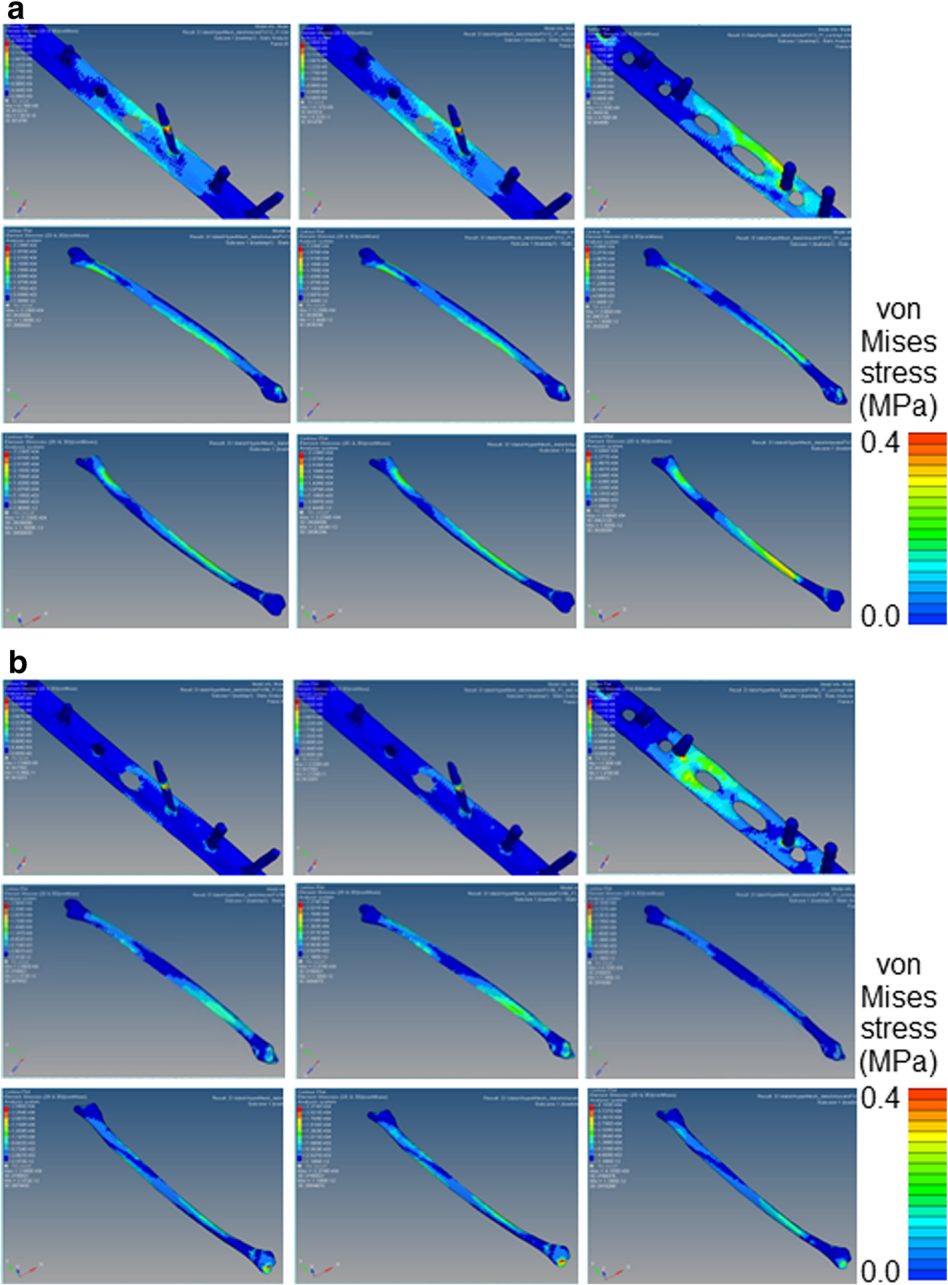
*Tops* are von Mises stresses on implant models of three internal fixations. *Middles* and *bottoms* are von Mises stresses on the fibula at lateral and medial sides. 1/3 TP + L + H (*right*). 1/3 TP + L (*mid*). LCP (*left*). **a** Mid stance. **b** Terminal stance

**Fig. 8:**
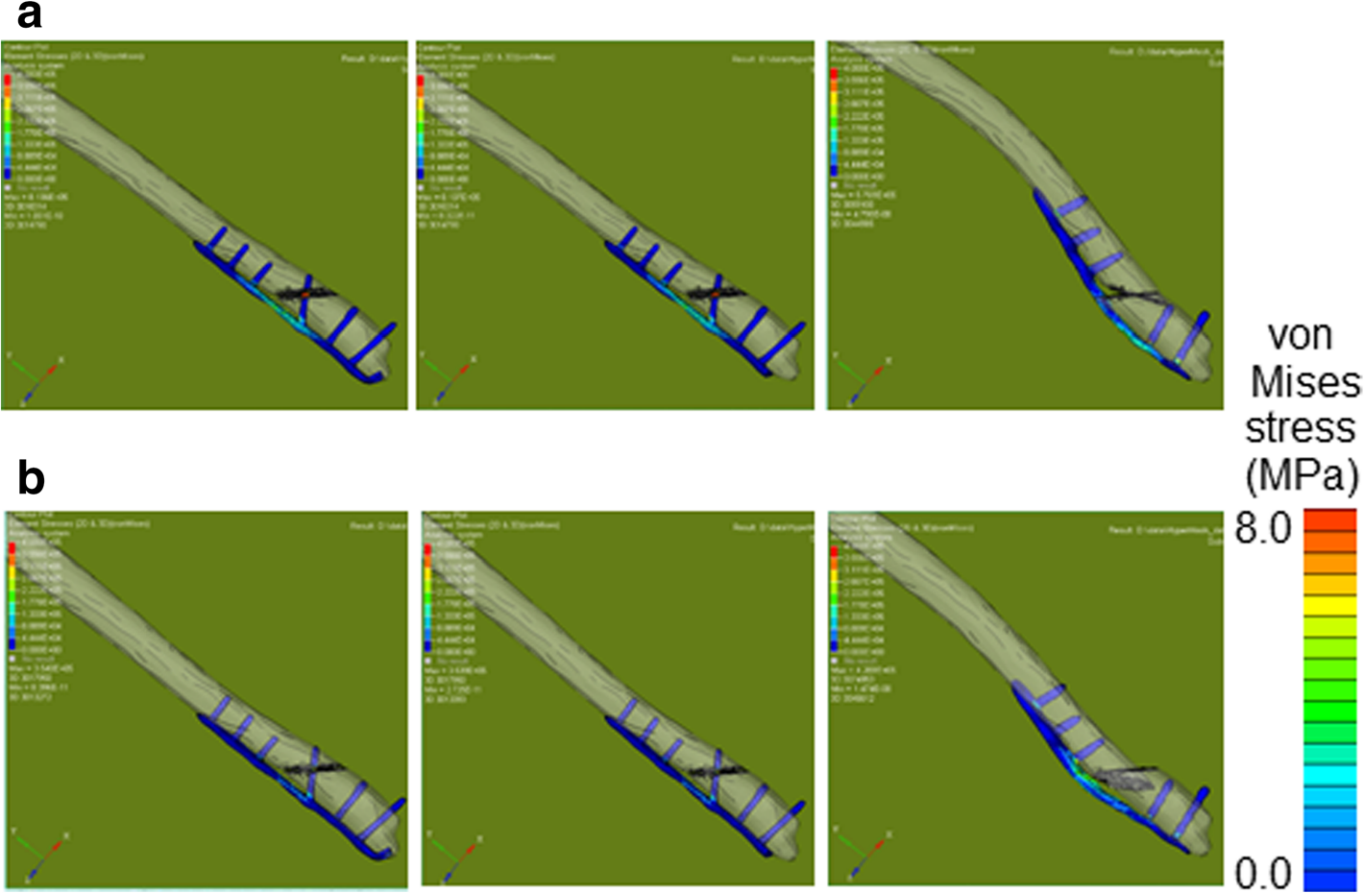
The scale of the von Mises stress was 20 times magnified to clarify the state of deformation. 1/3 TP + L + H (*right*), 1/3 TP + L (*mid*), and LCP (*left*). **a** Mid stance. **b** Terminal stance

On FEA, migration length between the fracture surfaces was more than two times longer in the LCP-fixed than in the one-third tubular plate-fixed bones (Fig. 5). The difference was marked on the medial side at TS, and migration length between the fracture surfaces fixed with LCP was five times longer than those in the other bones. Migration was not affected by the presence or absence of the hook. Displacement of the one-third tubular plate was small at both MS and TS regardless of the presence or absence of the hook compared with those of LCP (Fig. 6). At TS, displacement near the fracture line was markedly larger in LCP than in the one-third tubular plates and was greater at the proximal than distal site in all bones. von Mises stress on the plate was highest in LCP at both MS and TS. A marked difference in stress between the one-third tubular plates and LCP was noted when the ankle joint reaction force was maximum. Maximum von Mises stress on the fibula fixed with the one-third tubular plate differed between those with and without the hook by 11% and was higher in the presence of the hook (Fig. 7). When the scale of von Mises stress was maginified 20-fold to clarify deformation, marked deformation was noted at the root of the second locking screw from the distal end in LCP, whereas no deformation was observed in the one-third tubular plates (Fig. 8).

## Discussion

The lateral malleolus plays an important role as a dynamic stabilizer of the ankle joint. Dislocation of the lateral malleolus produces incongruence between the talus and ankle joint. Because this may cause secondary osteoarthritis, lateral malleolar fracture should be reduced and stabilized by internal plate fixation [1]. Many favorable treatment outcomes by internal fixation with LCP and one-third tubular plates have been reported [7, 8]. Hirakawa et al. reported that the time required for union was significantly shortened by the use of the hook, but no significant difference in clinical outcome was noted (JSSF ankle/foot scale) [8].

Stiffness of the one-third tubular plates was equivalent regardless of the presence or absence of the hook, and no significant difference was noted in the biomechanical study, suggesting that active preparation of the hook is unnecessary. Since the fixation stability of the hook is considerably smaller than those of the plates and screws, its presence or absence may have had no impact on stiffness. Another reason for the equal stiffness of the one-third tubular plates with and without the hook may have been the major influence of the lag screws. The fixation ability of the five cortical screws was inferior to that of the locking screws, but the biomechanical study suggested that stiffness equivalent to that of five locking screws can be acquired by the addition of a single lag screw. We assumed that displacement in the control with no fracture line was inhibited against loads in the bone axis direction compared with those in the other specimens, resulting in a higher value, although the difference was not significant. Torsional stiffness of the control specimen was lower than that of the others, suggesting that stiffness of the body part of the plate was effective.

The gait cycle is divided into the stance phase, during which the foot remains in contact with the ground, and the swing phase, during which the foot is not in contact with the ground [21]. The stance and swing phases are divided into five and three sequences, respectively. Among Frames 1–200 recorded during walking on the ground reaction force plates, we focused on four sequences in which characteristic changes were observed in gait, as follows. In the sequence observed in Frame 92, the right foot was in contact with the ground. This was regarded as the initial contact, and plantar flexion of the ankle joint was estimated to be 0° [21]. In the sequence observed in Frame 112, the knee joint reaction force reached maximum. This was regarded as MS, and dorsiflexion of the ankle joint was estimated to be 5°. In Frame 156, the ankle joint reaction force reached maximum. This was assumed to be TS, and dorsiflexion was estimated to be 10°. In Frame 174, the right foot was immediately before leaving the ground. This was regarded as pre-swing, and plantar flexion was judged as 15°. It was suggested that the sequence in which the force and torque loaded on the ankle joint reach maximum was that in which the dorsiflexion angle of the ankle joint reached the maximum. It has been reported that a weight about two–three times higher than the body weight is loaded on the lower limb during walking, suggesting that the results of the ergonomic study were valid [22]. When the load was calculated from the compressive stiffness calculated in the biomechanical study, it was found to be about two times higher than the body weight, and the reported value was covered on both approaches. The LCP specimen showed the high stiffness values, of which torsional stiffness was highest. LCP also showed higher displacement and migration length values. These findings suggest that the fracture region behaves differently between static and dynamic loading. Values may change depending on whether the patient is lying or walking. It would be interesting to evaluate the influence of this effect over a prolonged period, but this could not be determined from the present data.

Migration length between fracture surfaces on FEA was higher in the bone fixed with the LCP than with the one-third tubular plates, possibly due to the effect of the lag screws. The lag screws prevented shifting of the fracture surfaces, which may in turn have prevented displacement of the one-third tubular plates. von Mises stress loading on the plate was highest in LCP, and was more marked at TS than MS, which may have been due to a large dorsiflexion angle at TS. Maximum von Mises stress loaded on the fibulas fixed with the one-third tubular plates increased by 11%, and it is possible that the hook functioned in fixation of the fibula. Although the stress was high at the root of the lag screw near the fracture line, no deformation was noted in the one-third tubular plates, whereas the LCP was markedly deformed, suggesting that the lag screws prevent deformation when a large load is applied.

To ensure the validity of analysis, we output the balances of external forces on the ergonomic study and FEA and used them to confirm the consistency of all three internal fixations. A maximum error of 1 kg was noted, but this difference was due to the fact that the bone surface in the FEA model was not necessarily consistent with the muscle attachment sites defined in the AnyBody Modeling System ver.6.0.4. A healthy person was selected as subject for the motion capture of gait, but selection of a patient with lateral malleolar fracture would have provided clinically useful information. However, it is difficult to acquire reproducible and representative gaits among subjects with fracture. We considered that a healthy subject was appropriate because reproducibility is required for collection of basic data. When lateral malleolar fracture is not simple and stability cannot be acquired using lag screws, the antiglide plate procedure applying the plate to the posterior region is used [23]. Favorable clinical outcomes with this procedure have been occasionally reported in Japan and other countries. We restricted the present investigation to lateral application to standardize conditions, but are planning to report a study on the antiglide procedure.

Several limitations of our study warrant mention. First, as a biomechanical study, we used simulated bone which did not reflect the anatomical structures, such as the distal tibiofibular ligament. Second, the control was not investigated as a group.

## Conclusions

In this comparison of 1/3 TP + L + H, 1/3 TP + L, and LCP, we found that all three fixation methods provided equivalent stiffness to that of healthy bone. The presence of the hook did not make any difference in stiffness, suggesting that active preparation of the hook is unnecessary. We also clarified that lag screws inhibit displacement.
